# Septal Myectomy in Pediatric Obstructive Hypertrophic Cardiomyopathy: A Systematic Review and Meta-analysis

**DOI:** 10.1007/s00246-025-04088-w

**Published:** 2025-11-17

**Authors:** Anelise Poluboiarinov Cappellaro, Luiz Felipe Costa de Almeida, Ronaldo Altenburg Odebrecht Curi Gismondi, Rafael Ayala, Myat Soe Thet, Joseph A. Dearani

**Affiliations:** 1 Centro Universitário Maurício de Nassau de Barreiras, Barreiras, Brazil; 2 Department of Medical Sciences, Universidade Federal Fluminense, Niterói, Brazil; 3 Department of Cardiology, Universidade Federal Fluminense, Niterói, Brazil; 4 Cardiovascular Surgery Department, Robert Bosch Hospital, Stuttgart, Germany; 5 Department of Surgery & Cancer, Imperial College London, South Kensington, UK; 6 Department of Cardiovascular Surgery, Mayo Clinic, Rochester, MN USA

**Keywords:** Septal myectomy, Obstructive hypertrophic cardiomyopathy, Children, Pediatric

## Abstract

Evidence regarding outcomes of septal myectomy in pediatric patients with obstructive hypertrophic cardiomyopathy (HOCM) remains limited. This meta-analysis aimed to assess surgical outcomes and long-term survival in this population. A systematic review was conducted using PubMed, Embase, Scopus, and Cochrane databases to identify studies evaluating septal myectomy in pediatric patients with obstructive HCM. Eight clinical trials comprising a total of 490 pediatric patients met the inclusion criteria. Among children undergoing septal myectomy for HOCM, the pooled early and late mortality rates were 3.3% and 8.5%, respectively. Concomitant mitral valve repair was performed in 7.3% of patients. Rates of complete atrioventricular block and permanent pacemaker implantation were 9.9% and 5.1%, respectively. The mean durations of intensive care and hospital stay were 34.6 h and 12.9 days. Postoperative wound infection and reoperation occurred in 3.4% and 6.4% of cases. Echocardiographic assessments demonstrated a marked reduction in LVOT gradient (from 86.9 to 16.2 mmHg) with preserved left ventricular ejection fraction. Mitral regurgitation decreased from 49.8 to 6.4%, and systolic anterior motion from 84.9 to 19.9%. Septal myectomy in pediatric patients with HOCM is a safe and effective procedure, associated with favorable survival and substantial symptomatic and hemodynamic improvement. However, results must be interpreted cautiously due to moderate-quality retrospective data.

**Supplementary Information:**

The online version contains supplementary material available at https://doi.org/10.1007/s00246-025-04088-w.

## Introduction

Obstructive hypertrophic cardiomyopathy (HOCM) is an autosomal dominant genetic disorder characterized by abnormal, often asymmetric, thickening of the left ventricular (LV) wall, frequently resulting in dynamic left ventricular outflow tract (LVOT) obstruction [1, 2]. This phenotypically heterogeneous condition most commonly arises from heterozygous mutations in sarcomeric protein genes, particularly MYH7, MYBPC3, and TNNT2, typically within familial contexts [3]. In contrast, non-sarcomeric variants are associated with inborn errors of metabolism, congenital malformation syndromes, and neuromuscular disorders [4].

HOCM can manifest at any age, but early onset is generally linked to a worse prognosis [5]. It affects approximately 1 in 200 adults and is a leading cause of sudden cardiac death in the young [6]. In children, although rare, HOCM often follows a more aggressive course, with higher mortality compared to adults, underscoring the importance of early diagnosis and specialized management in this population [7].

Surgical septal myectomy is well-established as the standard treatment for symptomatic HOCM in adults [2]. On the other hand, pediatric HOCM is still a poorly explored area, with many gaps and limited consistent data available [8]. The existing literature on pediatric HOCM is often limited by incomplete reporting of critical outcomes such as survival, reoperation rates, and echocardiographic follow-up [8]. This absence of comprehensive data not only limits our understanding of disease progression but also complicates the development of standardized, evidence-based treatment protocols.

In response to these critical knowledge gaps, this study undertakes a systematic review and meta-analysis centered on pediatric patients with HOCM. By gathering the available evidence, we aim to provide more consistent data on surgical outcomes and survival rates. Our goal is to inform clinical decision-making and contribute to the development of evidence-based care for this patient population, where treatment options are still not fully established.

## Methods

The systematic review with meta-analysis was conducted in alignment with the Cochrane Collaboration Guidelines and the Preferred Reporting Items for Systematic Reviews and Meta-Analyses (PRISMA) guidelines [9, 10]. The study protocol was registered in the International Prospective Register of Systematic Reviews (PROSPERO) under the registration number (CRD420251044471).

### Objectives and Outcomes

This study aims to comprehensively evaluate the mortality rates and therapeutic efficacy of septal myectomy in pediatric patients (< 18 years) diagnosed with HOCM. Early mortality refers to deaths within 30 days post-surgery or during the initial hospital stay, while late mortality refers to deaths occurring thereafter, with follow-up extending up to several years. Secondary outcomes include the incidence of reoperation, postoperative complications, and changes in echocardiographic parameters before and after surgery.

### Search Strategy

We systematically reviewed the Medline (via PubMed), Cochrane, Embase, and Scopus databases in January 2025. Citations were extracted from each database and compiled using specialized systematic review software (Rayyan). Two independent authors blindly screened the citations based on predefined eligibility criteria, with a third author reviewing the results. Any discrepancies were resolved by consensus among three authors. The search strategy incorporated terms such as “children,” “obstructive hypertrophic cardiomyopathy,” “septal myectomy,” and their synonyms. The search strategy is available in Table S2.

### Eligibility Criteria

Studies were eligible for inclusion if they met the following criteria: [1] pediatric population (< 18 years), [2] diagnosis of obstructive hypertrophic cardiomyopathy, and [3] treatment with septal myectomy. Studies that did not meet these criteria, those that were not published in English, or lacked sufficient data for analysis were excluded. In studies with two arms, only the relevant arm was considered. No restrictions were applied based on sample size, publication date, or full-text availability. Study eligibility was not limited by follow-up duration to ensure the inclusion of both short- and long-term outcomes.

### Data Extraction

Two authors independently extracted data using a predesigned spreadsheet, capturing the following information: first author, year of publication, clinical and pathological patient characteristics, number of patients, follow-up duration, and relevant outcome data. A third author reviewed the extracted data, and a consensus resolved discrepancies.

### Quality Assessment

The risk of bias assessment was independently conducted by two authors using the Newcastle-Ottawa Scale (NOS), a validated tool specifically designed to evaluate the quality of non-randomized studies such as cohort or case-control designs. This scale examines how well each study was designed and executed, focusing on key methodological domains including the representativeness of the exposed cohort, selection of the non-exposed cohort, ascertainment of exposure, confirmation that the outcome was absent at baseline, outcome assessment, follow-up duration, and adequacy of follow-up. The NOS applies a semiquantitative “star system” across three domains: selection (up to 4 stars), comparability (up to 2 stars), and outcome/exposure assessment (up to 3 stars), with a maximum total score of 9. Higher scores reflect stronger methodological quality and more reliable evidence. In our analysis, studies were classified as follows: scores between 7 and 9 indicated a low risk of bias, scores between 4 and 6 indicated a moderate risk, and scores below 4 indicated a high risk of bias. By evaluating these aspects, the NOS captures potential weaknesses related to selection bias, comparability between groups, and reliability of outcome measurement.

### Statistical Analysis

 To combine and synthesize the results from the included studies, the metaprop function was applied to aggregate proportions, such as incidence or prevalence rates, calculating a weighted average of the individual study proportions based on sample size and precision. This approach allows for incorporation of variability between studies, reflecting differences in sample characteristics and estimates across investigations. For continuous outcomes, such as length of hospital stay or age, the pool.median function was used, enabling the integration of medians from different studies, even when reported in different formats (e.g., medians rather than means), while adjusting for sample size and data dispersion in each study. In all analyses, a random-effects model was applied to calculate 95% confidence intervals (95% CI), ensuring that both uncertainty and heterogeneity between studies were appropriately accounted for. All these functions are available in the R packages meta, metafor, and meta.median, which are widely used tools for conducting meta-analyses.

Heterogeneity among studies was quantified using the I^2^ statistic and further evaluated through the chi-square test (χ^2^), with statistical significance determined by the corresponding p-value.All statistical analyses for this systematic review were performed using R software (version 3.6.1).

## Results

### Baseline characteristics of included studies

**Fig. 1:**
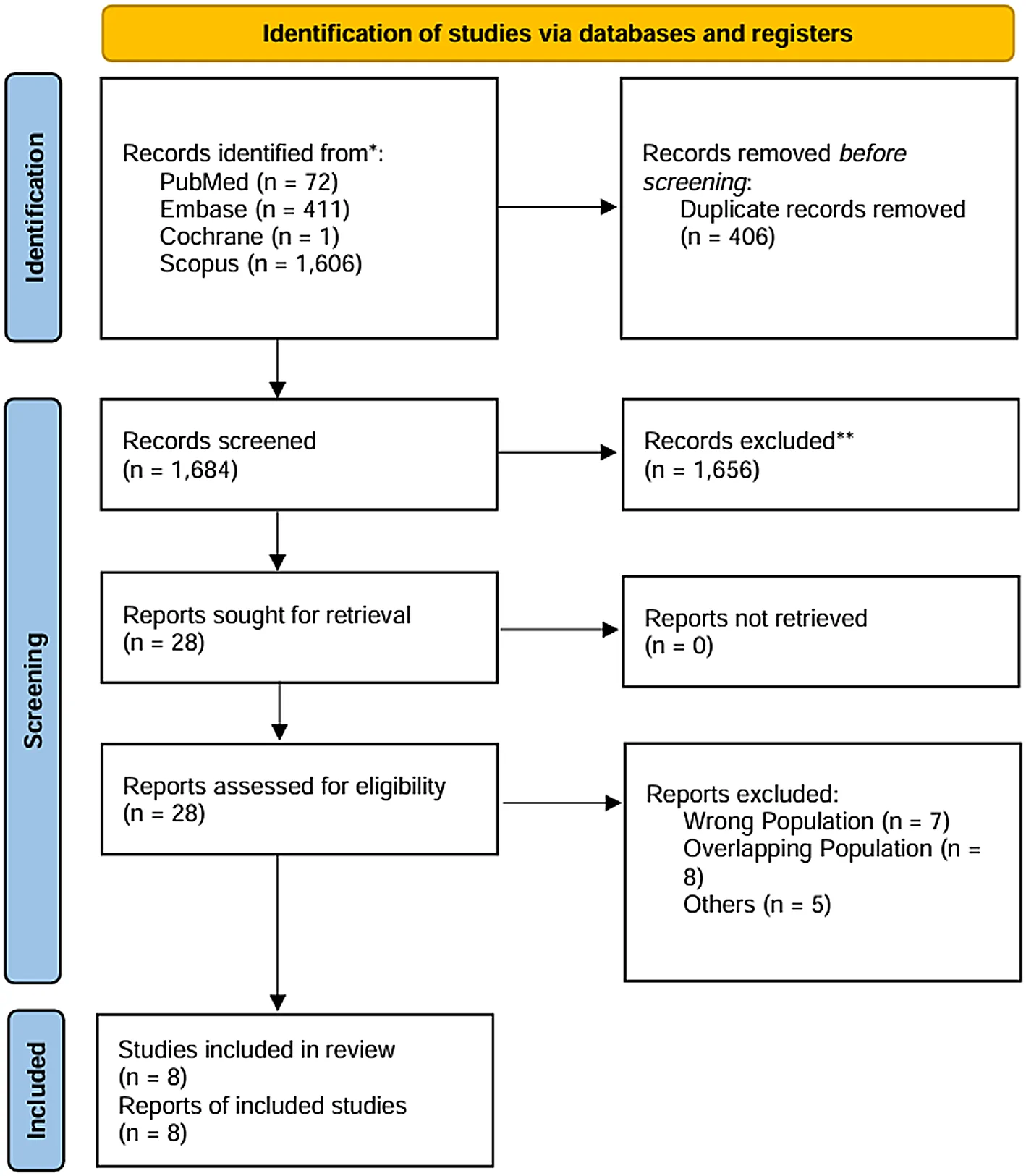
PRISMA flow chart. Preferred Reporting Items for Systematic Reviews and Meta-Analyses (PRISMA) flow diagram for literature search and selection

As illustrated in Fig. 1, the initial systematic search across PubMed, Embase, Scopus, and the Cochrane Library retrieved 2,090 records. Following the removal of duplicates and the screening of titles and abstracts for relevance, 28 full-text articles were assessed for eligibility. Ultimately, eight studies were included in the final analysis, comprising a total of 490 pediatric patients who underwent septal myectomy [11–18].

When multiple publications came from the same institution and involved overlapping patient populations, we included only the most recent and comprehensive study. For the Mayo Clinic (USA), only Griffeth (2023) was retained, while studies by Altarabsheh (2013), Menon (2008), Minakata (2005), Poterucha (2015), and Theodoro (1996) were excluded. Similarly, from Fuwai Hospital (China), Dong (2023) was selected over overlapping studies by Chen (2022), Xu (2016), and Zhu (2020).

The selected studies included patients from 1963 to 2020. Most were conducted at high-volume tertiary centers, such as the Mayo Clinic (USA), the German Heart Centre Munich (Germany), and Fuwai Hospital (China). All patients in the studies underwent transaortic myectomy, except in the Mayo Clinic cohort reported by Griffith et al., where 163 (82%) had transaortic, 16 (8%) transapical, and 19 (10%) a combined approach. The mean age of the patients ranged from 5.3 to 12 years, with a predominance of male patients in all studies. A family history of hypertrophic cardiomyopathy (HCM) or sudden cardiac death (SCD) was frequently reported. Additionally, Noonan syndrome was observed in several cohorts. These data are presented in Table 1. Main criteria for surgery were symptomatic refractoriness, hemodynamic severity, and gradient magnitude (see Supplementary Table S3 for study-specific details).

**Table 1:** Baseline characteristics of included studies

Studies	Center experience	Country	Design	Period inclusion	No. of Patients	Sex (M/F)	Mean age	Family history (HCM/SCD)	Noonan syndrome
Abdullah 2017	Kasr AlAini Hospitals, Cairo University	Egypt	Prospective observational study	2014–2016	9	7/2	6.1 ± 2.6	2	0
Dong 2023	Fuwai Hospital	China	Retrospective cohort	2013–2020	56	29/27	5.38 ± 3.78	6	12
Griffeth 2023	Mayo Clinic	USA	Retrospective cohort	1976–2021	199	N/A	12 ± 5.23	122	10
Hickey 2012	The Hospital for Sick Children	Canada	Retrospective cohort	1971–2006	32	N/A	N/A	14	N/A
Nguyen 2023	New York-Presbyterian/Morgan Stanley Children’s Hospital	USA	Retrospective cohort	1992–2022	37	23/14	7.9 ± 7.33	10	13
Schleihauf 2017	German Heart Centre Munich	German	Retrospective cohort	1978–2015	23	14/9	N/A	N/A	9
Stone 1993	National Heart, Lung and Blood Institute	USA	Retrospective cohort	1963–1987	17	10/7	11.9 ± 1.3	N/A	N/A
Zhu 2020	Fuwai Hospital	China	Retrospective cohort	2009–2018	117	75/42	11.3 ± 4.7	19	N/A

*USA* United States of America, *HCM* hypertrophic cardiomyopathy, Septal; *SCD* sudden cardiac death, *M* male, *F* female

**Table 2:** Echocardiographic indices preoperative and postoperative septal myomectomy in pediatric obstructive hypertrophic cardiomyopathy

Outcomes	Preoperative	Postoperative
Systolic anterior motion	84.93% (76.09%–94.80%)	19.91% (9.41%–42.09%)
Mitral regurgitation (≥ moderate)	49.77% (38.42%–64.47%)	6.40% (1.88%–21.80%)
LVOT peak gradient, mmHg (mean, range)	86.98 (80.53–93.42)	16.20 (13.50-18.91)
LVEF (%) (mean, range)	75.59 (71.05–80.14)	69.39 (63.75–75.03)

Changes in echocardiographic parameters before and after septal myectomy in pediatric obstructive hypertrophic cardiomyopathy

Significant reductions were observed in systolic anterior motion (SAM), ≥ moderate mitral regurgitation, and left ventricular outflow tract (LVOT) peak gradient, with a slight decrease in left ventricular ejection fraction (LVEF). Values are presented as mean (range) or percentage (range)

## Quality Assessment

The risk of bias was evaluated using the Newcastle–Ottawa Scale (NOS) for cohort studies. All included studies were classified as having moderate methodological quality, with NOS scores ranging from 5 to 6. The most common source of bias was in the Comparability domain, primarily due to insufficient control for potential confounding variables. Additionally, several studies did not fully meet the criteria within the Selection domain, particularly in terms of the representativeness of the exposed cohort and the adequacy of exposure ascertainment. In the Outcome domain, some studies failed to achieve maximum scores due to limitations in follow-up duration or the methods used for outcome assessment (Supplementary Table S4).

### Postoperative Surgical Outcomes

In pediatric patients with hypertrophic obstructive cardiomyopathy (HOCM) undergoing septal myectomy, the pooled early mortality rate was 3.27% (95% CI 1.76–6.07), whereas late mortality reached 8.49% (95% CI 3.86–18.67). The pooled incidence of concomitant mitral valve repair was 7.25% (95% CI 4.78–11.00). Persistent patent foramen ovale (PFO) following surgery was reported in 13.4% of patients (95% CI 6.48–27.71), while new iatrogenic ventricular septal defects occurred in 2.38% of cases (95% CI 0.69–8.20). Notably, wide confidence intervals observed for late mortality, persistent PFO, and iatrogenic ventricular septal defects highlight the imprecision inherent to the limited number of studies and patient heterogeneity.

The pooled mean aortic cross-clamp time was 54.2 min (95% CI 40.01–68.40), and the mean cardiopulmonary bypass duration was 85.6 min (95% CI 63.91–107.46). Complete atrioventricular block occurred in 9.86% of patients (95% CI 5.31–18.31), and left bundle branch block was observed in 27.75% (95% CI 20.82–36.98). Permanent pacemaker implantation was required in 5.13% of cases (95% CI 2.15–12.24). Pooled estimates for aortic cross-clamp and cardiopulmonary bypass durations, as well as conduction disturbances, are characterized by wide confidence intervals, reflecting both inter-study variability and limited sample sizes.

The incidence of postoperative wound infection was 3.35% (95% CI 1.17–9.59), and reoperation was necessary in 6.44% of patients (95% CI 4.17–9.93). The mean intensive care unit (ICU) stay was 34.6 h (95% CI 1.96–67.41), and the mean total postoperative hospital length of stay was 12.9 days (95% CI 6.01–19.89). Wide confidence intervals for postoperative outcomes, including wound infection, reoperation, ICU, and total hospital stay, indicate substantial heterogeneity and imprecision.

### Echocardiographic Outcomes

The preoperative peak left ventricular outflow tract (LVOT) gradient was markedly elevated, with a mean pressure of 86.9 mmHg (95% CI 80.53–93.43), and showed a substantial reduction following surgery to 16.2 mmHg (95% CI 13.50–18.91), reflecting significant hemodynamic improvement. Left ventricular ejection fraction (LVEF) was within normal limits prior to the procedure, averaging 75.5% (95% CI 71.05–80.14), and remained preserved or mildly decreased postoperatively at 69.3% (95% CI 63.75–75.04). The prevalence of mitral regurgitation (≥ moderate) before surgery was 49.77% (95% CI 38.42–64.47), which declined markedly to 6.4% postoperatively (95% CI 1.88–21.80). Similarly, systolic anterior motion was present in 84.93% of patients preoperatively (95% CI 76.09–97.80), with a notable postoperative decrease to 19.9% (95% CI 9.41–42.09).

**Fig. 2:**
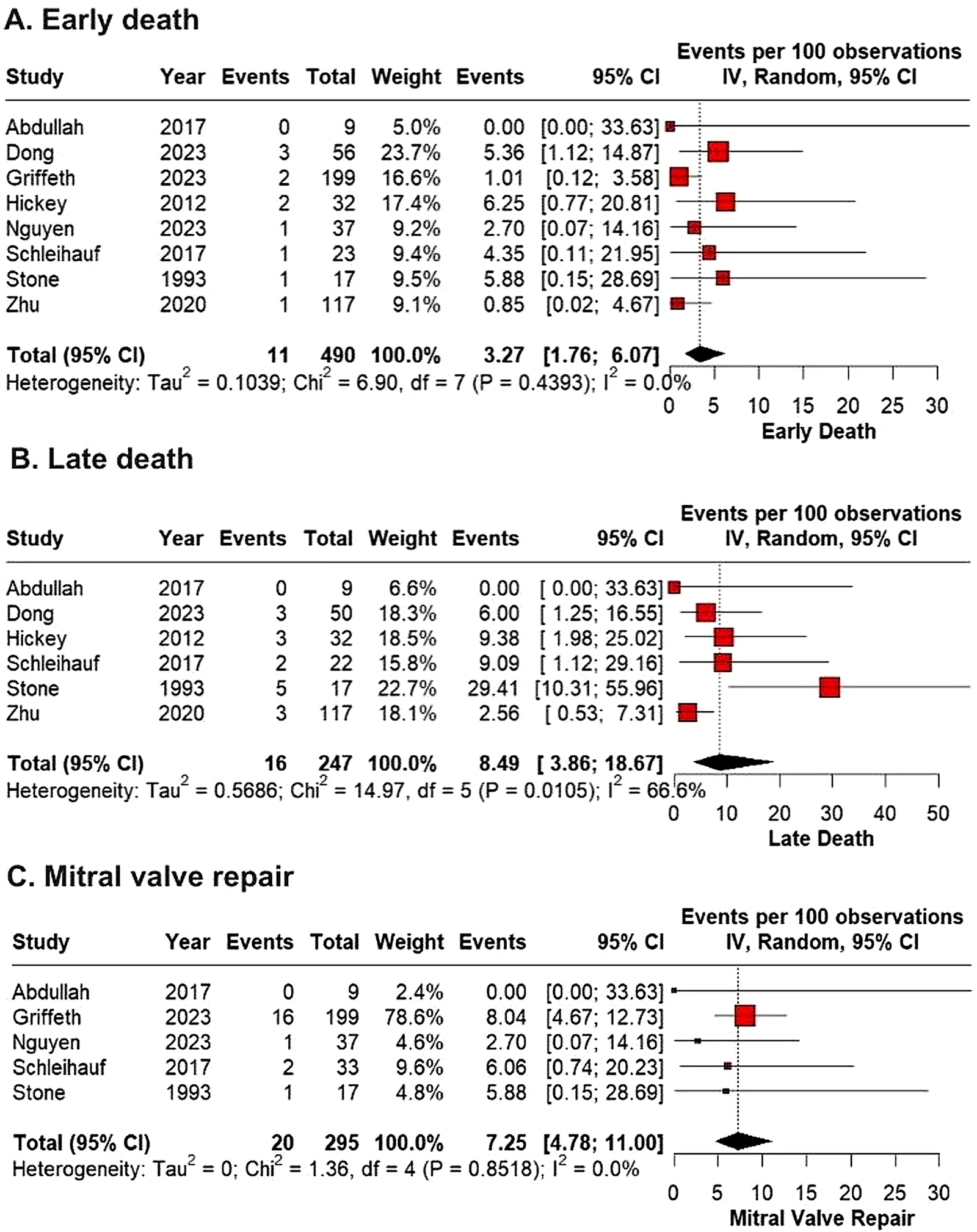
Forest plots of postoperative complications following septal myectomy in pediatric patients with obstructive hypertrophic cardiomyopathy. Horizontal lines through the squares depict 95% confidence intervals (CIs), indicating the range of uncertainty around each estimate. The diamond at the bottom of each plot represents the overall pooled estimate, with its width reflecting the 95% CI

## Discussion

This systematic review and meta-analysis of eight studies, encompassing 490 pediatric patients with HOCM who underwent septal myectomy, identified four key findings. First, the pooled estimates of early and late mortality rates establish crucial benchmarks for the safety of this procedure in a vulnerable patient population. Second, the procedure was consistently associated with a significant reduction in the LVOT gradient, highlighting its role in alleviating dynamic obstruction. Third, postoperative reductions in mitral regurgitation were commonly observed, suggesting beneficial remodeling of the mitral apparatus after septal decompression. Finally, improvement in SAM of the mitral valve was consistently reported, supporting the mechanical effectiveness of myectomy in addressing the key pathophysiological components of obstructive HOCM in children.

Septal myectomy remains the gold-standard surgical approach for patients with obstructive HOCM refractory to medical therapy [2]. The most used technique, the modified Morrow procedure, is typically performed via median sternotomy with intraoperative transesophageal echocardiographic guidance [19]. This technique involves longitudinal resection of the hypertrophied interventricular septum, extending beyond the basal segment toward the papillary muscles and, when necessary, into the apical septum [19]. This extended resection not only enlarges the LVOT but also directly addresses the anatomical substrate responsible for obstruction. Sustained reduction in LVOT gradient and restoration of near-normal intracardiac flow are critical for the long-term success of the procedure [19]. In cases with associated valvular or subvalvular anomalies, procedures such as the Konno-Rastan or Ross-Konno may provide broader anatomical correction [20]. As these techniques evolve, comparative studies will be essential to validate their safety and long-term efficacy in children.

Mortality in pediatric HOCM is influenced by a complex interplay of clinical and genetic factors [14]. Our findings align with prior evidence suggesting improved survival following myectomy in patients with obstructive physiology [21]. However, worse outcomes have been reported in younger children, particularly those with inborn errors of metabolism or restrictive phenotypes, as noted in multicenter registries [5]. These differences underscore the phenotypic heterogeneity of pediatric HOCM and the importance of individualized risk stratification. Echocardiographic parameters—including LVOT gradient, SAM, and left ventricular ejection fraction—remain central to diagnosis, surgical planning, and longitudinal follow-up [13, 21]. Improvements or stabilization in these metrics following myectomy have consistently been linked to better long-term outcomes [13].

The pharmacological treatment of HOCM in adults has traditionally relied on non-specific drugs, primarily aimed at symptom relief [2]. In refractory cases, invasive approaches such as septal myectomy and alcohol ablation are commonly used. More recently, in the EXPLORER-HCM trial (NCT03470545), cardiac myosin inhibitors have emerged as a promising targeted therapy by directly addressing the underlying pathophysiology of the disease and demonstrating significant improvements in LVOT obstruction and symptom relief [22]. The VALOR-HCM trial (NCT04349072) further showed that mavacamten may represent a viable non-invasive alternative for patients eligible for septal reduction [23]. In the pediatric population, mavacamten is currently being evaluated in the phase 3 randomized clinical trial SCOUT-HCM (NCT06253221) in adolescents with symptomatic HOCM [24]. While the results of this study are still pending, septal reduction through invasive intervention remains a viable treatment option for this group. Our findings provide supporting evidence that this approach is safe and may offer effective symptom relief.

### Limitations

This study offers strengths and limitations. First, variability in age distribution and patient phenotype across studies limits the applicability of pooled results, particularly to infants, adolescents, and syndromic or metabolic cardiomyopathies, where dedicated analyses remain lacking. Additionally, the analysis focused largely on echocardiographic and surgical outcomes, with less attention to functional aspects like quality of life andneurodevelopment. Several pooled outcomes, such as ICU stay (34.6 h, 95% CI 1.96–67.41), have wide confidence intervals, reflecting inter-study heterogeneity and limited sample sizes, which limits the ability to predict individual patient outcomes reliably. Furthermore, with only eight studies, each from a different center and spanning diverse inclusion periods and surgical eras, meaningful subgroup analyses were not feasible, and pooling such heterogeneous data may risk misleading conclusions.

Additionally, another limitation concerns the generalizability of our findings. All included studies were conducted in high-volume tertiary centers with extensive surgical expertise, which may not reflect outcomes in lower-volume or less specialized settings. Therefore, results in other institutions may differ, and validation through multi-institutional registries would be valuable to confirm the applicability of these findings more broadly. Most of the included studies focused on echocardiographic and surgical outcomes, while functional measures such as exercise capacity, neurodevelopment, and quality of life were not consistently reported. Despite being relatively large for this rare condition, the sample remains limited, and all studies were retrospective single-arm designs of moderate quality, warranting cautious interpretation of the findings.

### Future Directions

In pediatric HCM, non-sarcomeric etiologies, including metabolic and syndromic disorders, are associated with higher early mortality and transplantation risk, underscoring trajectories distinct from adults. Management should integrate genetic evaluation, CMR phenotyping, and pediatric-specific risk tools, while acknowledging their limitations. Although septal myectomy provides sustained relief of obstruction, many patients continue to experience heart failure, arrhythmias, or recurrent obstruction, highlighting the need for longitudinal, multidimensional follow-up that considers growth, neurodevelopment, and structured transition to adulthood.

Future research should prioritize patient-centered outcomes—including quality of life, daily functioning, and long-term neurodevelopment—while integrating genotype, phenotype, and extended follow-up. Such studies will guide more precise clinical and surgical decision-making and help develop pediatric-tailored strategies for optimizing care.

## Conclusion

This meta-analysis demonstrates that septal myectomy significantly improves hemodynamic parameters and symptom burden in pediatric patients with obstructive HOCM. However, these findings should be interpreted with caution, as the included studies were retrospective and of moderate quality. Long-term safety and effectiveness remain unproven, highlighting the need for prospective, multicenter registries incorporating functional and patient-centered outcomes to guide risk stratification and optimize individualized surgical strategies.

## Supplementary Information

Below is the link to the electronic supplementary material.

Supplementary Material 1 (DOXC 52 kb)

## Data Availability

No datasets were generated or analysed during the current study.
